# Efficient functional neutralization of lethal peptide toxins *in vivo* by oligonucleotides

**DOI:** 10.1038/s41598-017-07554-5

**Published:** 2017-08-03

**Authors:** Tarek Mohamed Abd El-Aziz, Corinne Ravelet, Jordi Molgo, Emmanuelle Fiore, Simon Pale, Muriel Amar, Sawsan Al-Khoury, Jérôme Dejeu, Mahmoud Fadl, Michel Ronjat, Germain Sotoing Taiwe, Denis Servent, Eric Peyrin, Michel De Waard

**Affiliations:** 1 INSERM UMR 1087/CNRS UMR 6291, Institute of Thorax, LabEx Ion Channels, Science and Therapeutics, 8 Quai Moncousu, BP 70721, 44007 Nantes Cedex 1, France; 2 University of Nantes, 44007 Nantes, France; 3 Zoology Department, Faculty of Science, Minia University, 61519 El-Minia, Egypt; 4 University Grenoble Alpes, 38000 Grenoble, France; 5 CNRS, DPM UMR 5063, 38041 Grenoble, France; 6 Service d’Ingénierie Moléculaire des Protéines (SIMOPRO), IBITECS, Commissariat à l’Energie Atomique, Université Paris-Saclay, F-91191 Gif sur Yvette, France; 7 Department of Zoology and Animal Physiology, Faculty of Sciences, University of Buea, P. O. Box 63, Buea, Cameroon; 8 CNRS, DCM UMR 5250, Bâtiment Nanobio, BP 53, 38041 Grenoble Cedex 9, France; 9 Smartox Biotechnology, 570 Rue de la Chimie, 38400 Saint-Martin d’Hères, France

## Abstract

Medical means to save the life of human patients affected by drug abuse, envenomation or critical poisoning are currently limited. While the compounds at risks are most often well identified, particularly for bioterrorism, chemical intervention to counteract the toxic effects of the ingested/injected compound(s) is restricted to the use of antibodies. Herein, we illustrate that DNA aptamers, targeted to block the pharmacophore of a poisonous compound, represent a fast-acting and reliable method of neutralization *in vivo* that possesses efficient and long-lasting life-saving properties. For this proof of concept, we used one putative bioweapon, αC-conotoxin PrXA, a marine snail ultrafast-killing paralytic toxin, to identify peptide-binding DNA aptamers. We illustrate that they can efficiently neutralize the toxin-induced (i) displacement of [^125^I]-α-bungarotoxin binding onto nicotinic receptors, (ii) inhibition of diaphragm muscle contraction, and (iii) lethality in mice. Our results demonstrate the preclinical value of DNA aptamers as fast-acting, safe and cheap antidotes to lethal toxins at risk of misuse in bioterrorism and offer hope for an alternative method than donor sera to treat cases of envenomation.

## Introduction

With more than 100,000 deaths and 400,000 amputations, envenomation has been added by the World Health Organization^1^ onto the list of tropical diseases neglected by global public health organizations^2^. Serum production, invented more than a century ago by Albert Calmette^3^, remains the only medical option for treating envenoming. It is however not the panacea. The serum is taken from horses or sheep that suffer from the venom injection steps. The immunoglobulin G has limited life shelf, often needs refrigerated storage which is a problem in tropical areas, and there are serious risks of anaphylactic and late adverse reactions. It should be emphasized that many toxins are either poorly or non-immunogenic, while some even induce immunosuppressive effects^4^. Furthermore, the immunoglobulin G, produced following whole venom injection, may lack selectivity for the most toxic components or, in addition, target compounds now considered to possess therapeutic value^5^. Finally, because of the restricted market size or the unaffordable cost of some antivenom vials ($2,000 for a single CroFab antivenom from BTG^6^), there is currently a dramatic shortage of antivenom supply^7^. Indeed, several traditional manufactures have been dropping out of the antivenom world market such as Behringerwerke AG (Germany), Sanofi Pasteur (France) and Commonwealth Serum Laboratories (Australia)^8, 9^.

Nevertheless, with the advent of venomics^10, 11^, a combination of modern transcriptomic and proteomic approaches, toxic venom components can now be isolated, identified and a toxicity score appended^12^. In some cases, the venom toxicity can even be appended to a single toxin^13^. The approach has the potential to rationalize any approach aimed at more efficiently neutralizing those toxins that pose a threat to human condition^14^. Notably, most venom toxins can now be chemically synthesized or made by recombinant expression, potentially circumventing the need to milk venoms. In view of these technological advances, we questioned whether it would be possible to replace the century-old immunoglobulin G production method to neutralize dangerous toxins by implementing a new rupture technology in toxinology. We turned towards functional oligonucleotides such as DNA aptamers for several reasons. Compared to antibodies (i) they have a small size, (ii) their production is easy, reproducible and cost-effective, (iii) they can easily be stored at ambient temperature, and (iv) they are poorly immunogenic and lack toxicity^15, 16^. In addition, they should be able to bind toxins for which no immune response can be triggered. Aptamers can easily be labeled with various tags at precise locations without altering their binding affinity. They have proved useful in numerous applications such as biosensors^17^, bio-imaging^18, 19^, nanotechnology^20^, delivery agents^18, 20^, diagnostic tools^21^ or therapeutic agents^22^. For proof of concept that aptamers have the ability to neutralize dangerous animal toxins, we focused on a fast-killing paralytic α-conotoxin^23, 24^. These compounds have been listed by the USA Center for Disease Control and Prevention as potential bioweapons in agreement with the Public Health Security and Bioterrorism Preparedness and Response Act of 2002^25, 26^. In spite of the risks of misuse of these compounds, no tailored antidotes exist for paralytic α-conotoxins^27^. α-conotoxins act very rapidly to produce paralysis indicating that they represent ideal and challenging cases of study. While this report is of interest to the field of toxinology, we also believe that it sets the trend for any poisonous compound that puts human life at risk, including drug abuse, phytochemicals or viral infection.

## Results

### Toxin lethality and aptamer selection

**Figure 1:**
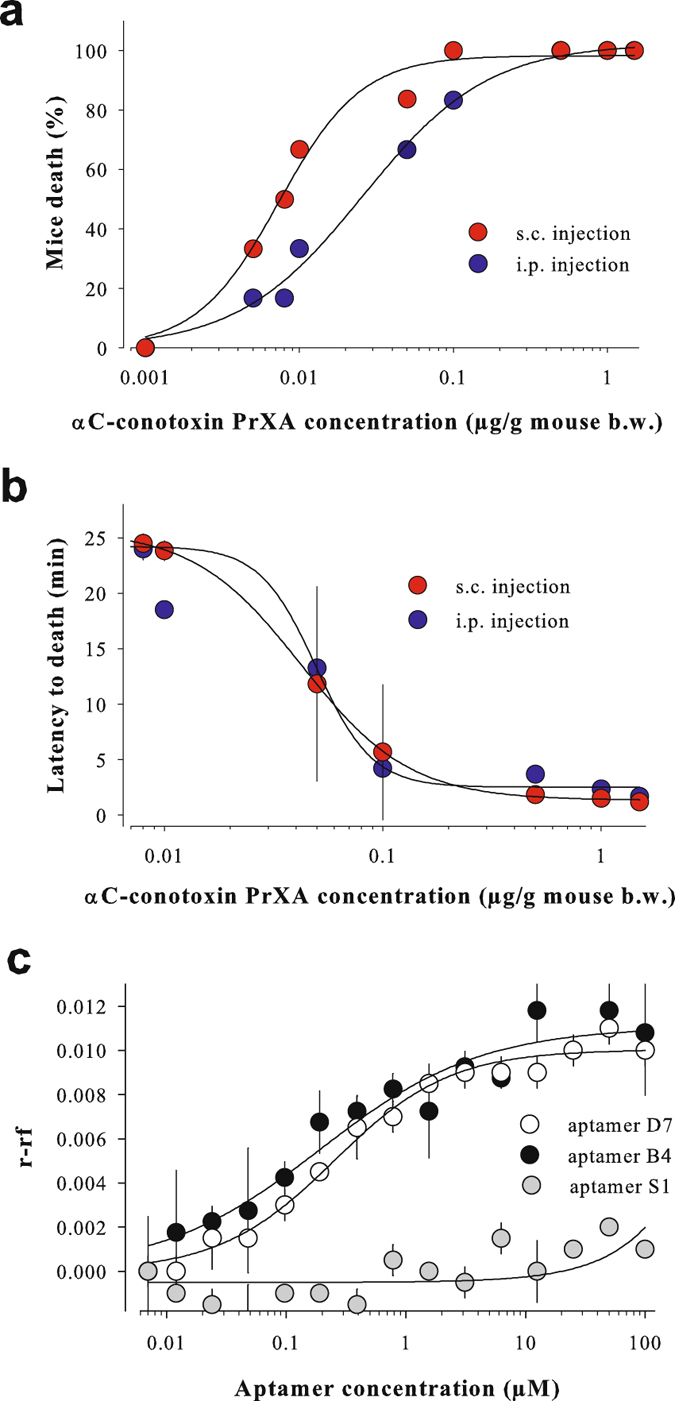
Toxin lethality and aptamer selection. (**a**) Dose-response curve illustrating αC-conotoxin PrXA-mediated mice death. i.p. and s.c. injection. (**b**) Shortening of the latency of death induction as a function of αC-conotoxin PrXA concentration. (**c**) Binding affinities of D7, B4 and S1 scramble aptamers towards 20 nM FAM-αC-conotoxin PrXA using fluorescence polarization. r: anisotropy of FAM-αC-conotoxin PrXA in presence of aptamer, rf: anisotropy of free FAM-αC-conotoxin PrXA.

We first chemically synthesized the 32 amino-acid and one disulfide-bridged αC-conotoxin PrXA from the fish-hunting marine snail *Conus parius* (Supplementary Fig. 1a). αC-conotoxin PrXA was selected over other α-conotoxins because it is highly potent and selective for the nicotinic receptor of skeletal muscles^28^. Next, we illustrated the fast killing aptitude of this toxin in *Mus musculus* Swiss mouse following two routes of administration (intraperitoneal (i.p.) and subcutaneous (s.c.)). αC-conotoxin PrXA-induced mouse lethality occurred with a LD_50_ of 0.027 ± 0.003 µg/g (i.p., n = 48) and 0.008 ± 0.001 µg/g mouse body weight (b.w.) (s.c., n = 48) (Fig. 1a). Maximal lethality was observed at 0.5 µg/g mouse b.w. (i.p.) and 0.1 µg/g mouse b.w. (s.c.). Toxin s.c. was thus a more efficient administration than i.p. to induce mice lethality. Increasing concentrations of the toxin shortened the delay for death occurrence (Fig. 1b). Toxin concentrations required to produce a twofold decrease in death latency were 0.050 ± 0.013 µg/g mouse b.w. (i.p.) and 0.043 ± 0.002 µg/g mouse b.w. (s.c.). Fastest death occurrence occurs within 2.4 ± 1.2 min (i.p.) or 1.5 ± 0.2 min (s.c.) following toxin administration. These data indicate that s.c. administration is better than i.p. to optimize the pharmacokinetics of toxin diffusion within mice. Death was preceded by a series of mouse phenotypic alterations (Supplementary Table 1). Next, a CE-SELEX procedure^29^ was used to identify single-stranded DNA (ssDNA) aptamers binding onto αC-conotoxin PrXA from a commercial random library of 10^12^ sequences (Supplementary Fig. 2). Several ssDNA aptamer sequences were identified from the fourth round of selection and sequence alignment revealed three distinct groups of homolog sequences (Supplementary Table 2). A total of six aptamers were randomly chosen from these groups: A5 and D3 (group 1), B4 and A4 (group 2), and B3 and D7 (group 3). Their dissociation constants were determined by measuring the change in fluorescence anisotropy of 5,6-carboxyfluorescein (FAM)-αC-conotoxin PrXA (synthesis described in Methods, Supplementary Fig. 1b) upon aptamer binding. Two representative examples are shown for the B4 and D7 aptamers that yielded K_d_ values of 120 ± 31 nM (B4) and 237 ± 30 nM (D7) (Fig. 1c). Proposed secondary structures for these two aptamers are provided (Supplementary Fig. 3). Globally, the following rank order of affinity was determined: B4 ≥ D3 > A5 > D7 ≥ A4 ≫ B3 with values ranging from 120 nM to more than 5 μM (Supplementary Table 3). No anisotropy signal variation was observed with the S1 scramble aptamer, confirming the selectivity of these interactions (Fig. 1c and Supplementary Table 3). Next, we used Surface Plasmon Resonance (SPR) technology to assess the ability of a toxin-selected aptamer to detect αC-conotoxin PrXA in solution. As shown on the sensorgrams, the 5′-biotinylated B4 aptamer, immobilized to saturation on the streptavidin sensor chip, dose-dependently detected αC-conotoxin PrXA (Supplementary Fig. 4). Time constants of association are linearly dependent on toxin concentration (7286.6 M^−1^.s^−1^), while dissociation time constant is concentration-independent (0.0024 s^−1^). The K_d_ of 329 nM is in close agreement with the 120 nM value determined by fluorescence anisotropy. These data illustrate that aptamers can be immobilized, remain able to detect the native label-free toxin and are sensitive compounds for detecting toxins at risks, confirming an earlier feasibility report^30^.

### *In vitro* evaluation of aptamer neutralization properties

**Figure 2:**
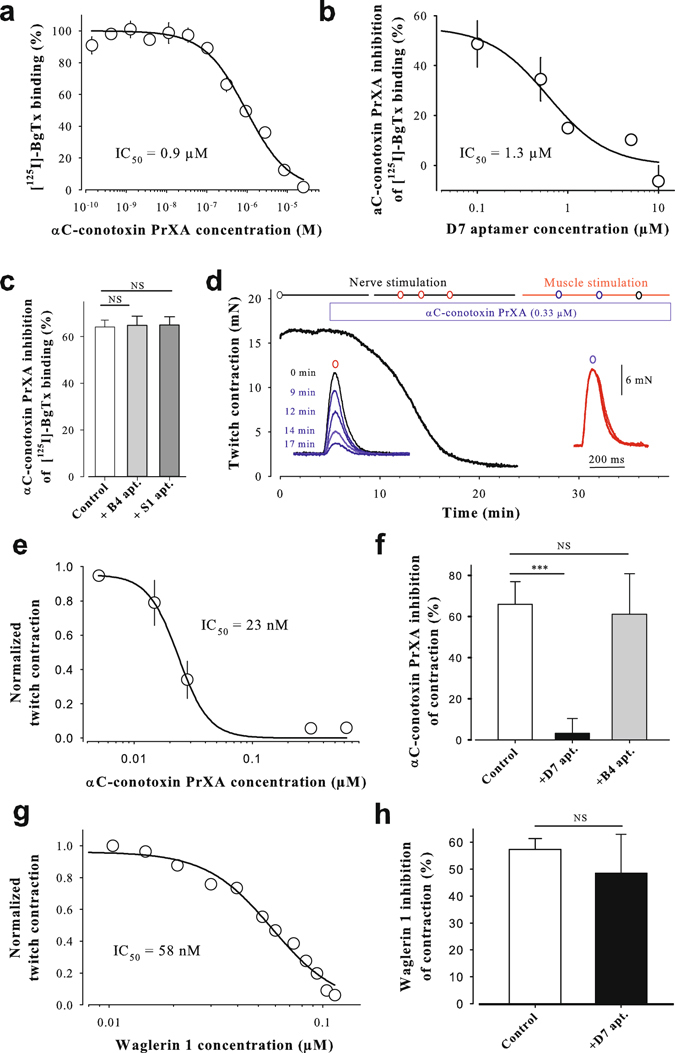
*In vitro* evaluation of aptamer neutralization properties. (**a**) Dose-response curve of αC-conotoxin PrXA-mediated inhibition of 1 nM [^125^I]-α-BgTx binding. (**b**) Effect of D7 aptamer on αC-conotoxin PrXA-mediated inhibition of [^125^I]-α-BgTx binding. (**c**) Effect of B4 aptamer on αC-conotoxin PrXA-mediated inhibition of [^125^I]-α-BgTx binding. (**d**) Time-dependent inhibition of muscle contraction by 0.31 µM αC-conotoxin PrXA (left inset). Lack of toxin effect on direct muscle stimulation (right inset). (**e**) αC-conotoxin PrXA dose-dependence of muscle contraction inhibition. IC_50_ = 23 nM. (**f**) Effect of D7 and B4 aptamers on 28 nM αC-conotoxin PrXA-mediated inhibition of muscle contraction. D7 at 20x molar, B4 at 45x molar concentration. (**g**) Waglerin 1 dose-dependence of muscle contraction inhibition. (**h**) Effect of D7 aptamer on waglerin 1-mediated inhibition of muscle contraction. N.S.: nonspecific; ***: p ≤ 0.001.

To get insight on the neutralizing properties of the aptamers, we first assessed the ability of αC-conotoxin PrXA to displace [^125^I]-α-BgTx binding^31, 32^ onto *Torpedo* muscle-type nicotinic acetylcholine receptors (nAChRs). The toxin fully inhibited [^125^I]-α-bungarotoxin (BgTx) binding with an IC_50_ of 0.9 ± 0.1 µM (Fig. 2a). Using the Cheng-Prusoff equation (1)

1 $$Ki=I{C}_{{\rm{50}}}/(1+({\rm{L}}\ast \frac{1}{{\rm{Kd}}}))$$

the toxin K_i_ value for binding to *Torpedo* nAChRs was 17 ± 2 nM. Next, we evaluated the effect of B4 and D7 aptamers on 1 µM αC-conotoxin PrXA-mediated inhibition of [^125^I]-α-BgTx binding. As shown in Fig. 2b, the D7 aptamer dose-dependently prevented the inhibition of [^125^I]-α-BgTx binding by αC-conotoxin PrXA with an IC_50_ of 1.3 ± 0.6 μM. Complete prevention occurred at 10 µM D7 aptamer. In contrast, the B4 and S1 aptamers lacked these neutralizing properties, demonstrating the specificity of the D7 aptamer (Fig. 2c). It is concluded that the D7 aptamer has the ability to neutralize the pharmacophore of αC-conotoxin PrXA. As this toxin acts on nAChR, it is predicted to be paralytic. Thus, we characterized the effect of αC-conotoxin PrXA on muscle contractions evoked by nerve stimulation in isolated mouse phrenic-hemidiaphragm nerve-muscle preparations. Application of 0.3–1 µM αC-conotoxin PrXA produced a concentration- and time-dependent inhibition of muscle contraction with full block occurring after 15 min with the highest toxin concentration used (Fig. 2d and left inset). The inhibition of contraction is the result of postsynaptic nAChR block and not a direct effect on the contraction machinery since direct electrical stimulation of the hemidiaphragm muscle did not alter muscle contractility force (Fig. 2d and right inset). The αC-conotoxin PrXA inhibited nerve-evoked muscle contractions with an IC_50_ of 23 ± 2 nM (Fig. 2e). Consistent with its effects on αC-conotoxin PrXA-mediated inhibition of [^125^I]-α-BgTx binding, 1.2 µM D7 aptamer produced complete block of contraction inhibition (Fig. 2f). In contrast, the B4 aptamer had no effect at 2.5x higher concentration (Fig. 2f). For complete assessment of selectivity, a venom snake nicotinic toxin blocker, waglerin 1^33^, was synthesized and tested for its inhibition of muscle contraction (Fig. 2g). Waglerin 1 produced full block of contraction with an IC_50_ = 58 ± 2 nM, which is twofold less potent as αC-conotoxin PrXA. As shown in Fig. 2h, the D7 aptamer did not affect the waglerin 1 activity, indicating its specificity towards αC-conotoxin PrXA.

### *In vivo* evaluation of aptamer neutralization properties

**Figure 3:**
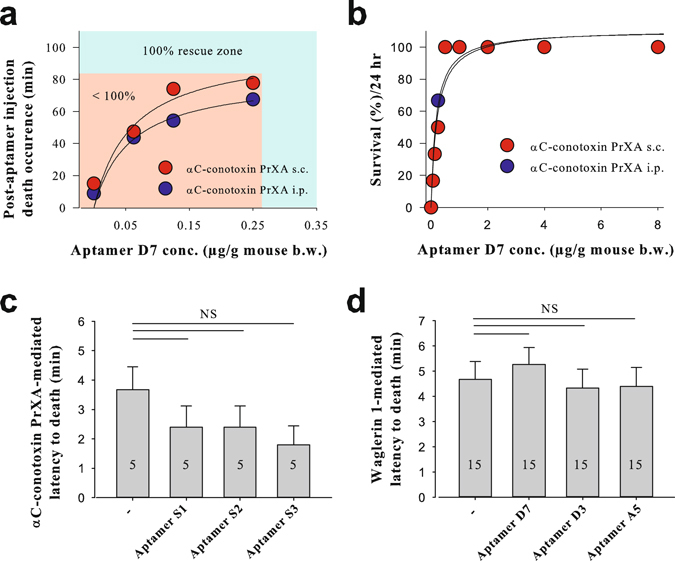
*In vivo* evaluation of aptamer neutralization properties. (**a**) Time of death of mice induced by 0.5 µg toxin/g mouse b.w. as a function of D7 aptamer concentration. t = 0 min toxin injection (s.c. or i.p.), t = 1 min aptamer injection (i.v.). (**b**) Mice survival as a function of i.v.-injected D7 aptamer concentration. (**c**) Lack of effect of scramble oligonucleotides on αC-conotoxin PrXA-mediated death. (**d**) Lack of effect of D7, D3 and A5 aptamers on waglerin 1-mediated mice death.

Because the D7 aptamer efficiently neutralizes αC-conotoxin PrXA *in vitro*, it was also tested for its ability to block the lethal effect of 0.5 µg toxin/g mouse b.w. (i.p. and s.c.). This toxin concentration was sufficient to induce 100% death of mice (Fig. 1a), regardless of the mode of administration, and within 3.7 ± 0.8 min (i.p.) or 1.8 ± 0.6 min (s.c.) (Fig. 1b). The toxin was injected i.p. or s.c. until the first symptoms of lethal intoxication developed. Next, various concentrations of D7 aptamers were injected 1 min later in the mouse tail vein (i.v.), a completely different body location. For low aptamer concentrations that did not rescue all mice from death, the delay between toxin injection and death occurrence could be measured on some individuals (Fig. 3a). As shown, i.v. injection of the D7 aptamer at concentrations up to 0.25 μg/g mouse b.w. had the potency to delay toxin-induced death occurrence up to 67.5 ± 1.5 min (i.p.) or 77.7 ± 2.4 min (s.c.). The concentration of D7 aptamer that increases the latency to death to half its maximal value was 0.06 ± 0.05 µg/g mouse b.w. (for toxin i.p.) and 0.06 ± 0.03 µg/g mouse b.w. (for toxin s.c.). The similarity of these two values indicates that the i.v.-injected D7 aptamer neutralizes the toxin with the same pharmacokinetics regardless of the mode of administration of the toxin (i.p. or s.c.). However, a slightly shortly longer time is needed to make sure that s.c.-toxin-injected mice can survive following neutralizing aptamer injection. Finally, at higher D7 aptamer concentrations, all mice survived the toxin injection regardless of the mode of toxin administration (Fig. 3b). Survival was examined over 24 h indicating that the aptamer neutralization has a durable effect. Also, no rebound effect could be observed up to one week after toxin administration. Concentrations of i.v.-injected D7 aptamer required to rescue 50% of the mice from toxin-induced death were 0.18 ± 0.05 µg/g (i.p.) and 0.22 ± 0.06 µg/ g mouse b.w. (s.c.), again indicating similar efficiencies of the aptamer in the i.p. and s.c. modes of toxin administration. Two types of controls were performed for investigating the selectivity of effect of the D7 aptamer. Three scrambled DNA sequences were without effect on αC-conotoxin PrXA-mediated death injection *in vivo* (Fig. 3c). Also, the D7, D3 and A5 aptamers, all recognizing αC-conotoxin PrXA, proved ineffective at protecting mice against death induced by 2.5 μg waglerin 1/g mouse b.w. (Fig. 3d), a concentration that induces 100% mice lethality within 4.7 ± 0.7 min (Supplementary Fig. 5).

## Discussion

In conclusion, we provide strong evidence that a subset of anti-toxin aptamers potently inhibits peptide pharmacology *in vitro*, presumably through the neutralization of the toxin pharmacophore. Evidence that DNA aptamers could neutralize toxins *in vitro* was also provided by a study illustrating *Staphylococcus aureus* α-toxin inhibition by four different aptamers^34^. Nevertheless, this study never extended to *in vivo* observations. In contrast, our report on the αC-conotoxin PrXA-neutralizing aptamer extents the observations to the preclinical level and leads to several important conclusions. First, i.v. injection of the aptamer is highly efficient in neutralizing αC-conotoxin PrXA in experimental conditions in which mice still have a limited time of survival: 2.7 min (i.p.) or 0.8 min (s.c.) post-aptamer injection, suggesting rapid diffusion in the organism, target recognition and neutralization in spite of a complex molecular and cellular environment. This finding nicely supports the general belief that aptamers have excellent tissue penetration. Second, this effect is long-lasting (>1 week) for aptamer concentrations that induce 100% mice rescue, demonstrating the stability of the complex that avoids the late reappearance of toxic effects. Alternatively, the aptamer neutralization time or aptamer half-life may be sufficient to effectively remove the toxin of the circulation by renal clearance. Third, for any concentration of aptamer, survival time beyond 78 min is a synonym of rescue. Considering the mechanism of toxin neutralization, the following may occur: (i) fast pharmacokinetic diffusion and specific target recognition, in agreement with earlier observations on aptamers^35^, (ii) block of the toxin pharmacophore, and (iii) possible alteration of the normal bio-distribution and pharmacokinetics of the toxin. Concerning the later, more work needs to be done by examining the impact of DNA aptamers on the *in vivo* biodistribution, stability and clearance pathways of imaging-compatible tagged versions of the toxin.

While we present a proof of concept that DNA aptamers work remarkably well in neutralizing a single lethal toxin, it is also reasonable to question ourselves on the reproducibility of this technique for αC-conotoxin PrXA itself and its applicability to related conotoxins or more largely to animal venoms as cocktails of different toxins. Concerning the first issue, it is fair enough to state that the chemical synthesis of various conotoxins is now perfectly mastered by various actors throughout the world and that the available technologies ensure that each synthetic compound is fully identical to its native counterpart. One important matter with conotoxins is often to make sure that the native disulfide bridge connectivity is well identified and properly reproduced during chemical synthesis as introducing a non-native disulfide bridge organization is susceptible to alter the conformation and pharmacology of the toxin^36^ and its recognition by the aptamer. Here again the technologies of disulfide bridge determination are mature enough so that this pitfall can be avoided properly. Besides αC-conotoxin PrXA contains a single disulfide bridge and no error in connectivity can be introduced during chemical synthesis in this particular case. No variability should therefore be expected at the level of conotoxin synthesis, be it at a small or a large production scale. Similarly, once their sequences are identified, aptamers can be commercially produced with both high yield and high reproducibility by proven-effective chemical solid-phase synthesis methods^37^. Of note, new technologies are emerging, such as PCR or asymmetric PCR, that will soon allow for unlimited and inexpensive production of DNA aptamers at the gram or large scale^38^. We ourselves used two different batches of the B4 aptamer for determining the dissociation constants by either fluorescence anisotropy or SPR and found very similar values, indicating minimal batch to batch variability. In our opinion, there is therefore no limitation in producing large quantities of DNA aptamers that reproducibly should neutralize synthetic and natural αC-conotoxin PrXA alike. Now, concerning the second issue on the applicability of the technique to related conotoxins or venoms, our methodology may require some adaptations to enlarge its neutralizing potential. The SELEX procedure allowed us to recover many different aptamer sequences that differ in sequence and structure and it is therefore likely that many of these aptamers, initially selected towards αC-conotoxin PrXA, may also recognize and neutralize structural variants of this toxin that differ by single amino acid substitutions. Alternatively, for conotoxin variants that differ more drastically in amino acid sequence, it is conceivable to perform the aptamer selection in such a way that the selected DNA aptamers recognize several conotoxin variants at a time. In the case of venoms, the number of toxins and diversity of sequences and structures encountered vary considerably and therefore the SELEX procedure should aim at identifying a cocktail of aptamers with the potential to neutralize several toxins at a time *in vivo*. Such a selection approach may of course be considerably eased by focusing only on the toxins that present lethal risks and simply parallelizing the approach we present herein. As such, our observations constitute the first preclinical proof of concept that functional oligonucleotides can be added to the list of antidote compounds available to counter the medical consequences of bioterrorism and envenomation.

## Materials and Methods

The *Materials* section and methods related to toxin chemical syntheses, aptamer selection using CE-SELEX, aptamer binding affinity measurements with surface plasmon resonance are all available in *SI Materials and Methods*.

### Aptamer binding affinity using fluorescence anisotropy

The binding affinity of the selected label-free aptamer sequences against αC-conotoxin PrXA was measured using fluorescence anisotropy on a Tecan Infinite F500 microplate reader (Männedorf, Switzerland), which offers excellent levels of sensitivity for fluorescence-based assays. The toxin was dissolved at 1 mM in dimethylformamide (DMF) and then diluted at 25 nM in TGK buffer containing 0.1% Tween 20. Initial concentrations of aptamers (200 µM) were prepared in TGK, heated to 80 °C for 5 min, and kept at room temperature for 30 min. Different concentrations of aptamers (0.012, 0.024, 0.048, 0.097, 0.19, 0.39, 0.78, 1.56, 3.125, 6.25, 12.5, 25, 50, 100 µM) were prepared by diluting the initial concentration with TGK buffer. In order to avoid toxin absorption onto the plastic wells, the microplate (Greiner 384 Flat Bottom Black plate, 10 µL) was washed two times with bovine serum albumin (BSA, 10 mg/1 mL of H_2_O) and one time with TGK buffer. The different concentrations of aptamers were loaded in duplicate with the FAM-labeled αC-conotoxin PrXA into the microplate wells (10 µL) and incubated for 60 min at 4 °C after which the fluorescence anisotropy values were measured. The fluorescence intensity of the toxin was monitored by exciting FAM-αC-conotoxin PrXA at 485 nm and measuring the emission at 520 nm. The change in anisotropy was the average anisotropy of the initial FAM-αC-conotoxin PrXA subtracted from the average anisotropy value at each aptamer concentration as previously reported for the analysis of adenosine–aptamer binding data^39^. The following modified one-phase exponential association equation (2) was employed to estimate the apparent dissociation constant K_d_:

2 $${\rm{\Delta }}{\rm{r}}=r-rf=(rb-rf)\times (1-{e}^{-kc})$$

where r is the measured anisotropy, rf the anisotropy in absence of aptamer, rb the anisotropy of maximally target-associated aptamer, c the concentration of target molecule and k a fit parameter. The nonlinear regression of the r *versus* c plots, where rb and k constitute the adjustable parameters, was achieved using the table curve 2D software (Systat Software Gmbh, Erkrath, Germany) and the apparent K_d_ value was determined from the midpoint of the equation, i.e.

$$-\mathrm{ln}(\frac{0.5}{k})$$

### Aptamer-mediated neutralization of αC-conotoxin PrXA-induced inhibition of isometric twitch tension from isolated mouse hemi-diaphragm

All animal procedures were conducted with the approval of the CEA Animal care and Ethics Committee; experimental protocols were approved by the French Departmental Direction of Animal Protection (n° A91-453). Adult Swiss mice (25–30 g) were euthanized by cervical dislocation followed by immediate exsanguination. Phrenic nerve hemi-diaphragm muscle preparations were isolated and mounted in a 4 mL capacity silicone-lined organ bath containing a Krebs-Ringer solution of the following composition: 140 mM NaCl, 5 mM KCl, 2 mM CaCl_2_, 1 mM MgCl_2_, 11 mM glucose, and 5 mM HEPES (pH 7.4), continuously superfused with pure O_2_ throughout the experiment (temperature 22 °C). For isometric twitch tension measurements, one of the hemi-diaphragm tendons (at the rib side) was firmly anchored onto the silicone-coated bath with stainless pins, and the other tendon (central medial tendon) was attached *via* an adjustable stainless-steel hook to an FT03 isometric force transducer (Grass Instruments, West Warwick, RI, USA). The resting tension was monitored and adjusted for each preparation tested with a mobile micrometer stage. The motor nerve of isolated neuromuscular preparations was stimulated *via* a suction electrode with pulses of 0.15 ms duration and supramaximal voltage (5–12 V) at a stimulation rate of 0.15 Hz, through the isolation unit of a square pulse S-48 stimulator (Grass Instruments, West Warwick, RI, USA). In some experiments, direct electrical muscle stimulations were performed with an electrode assembly placed along the length of the hemi-diaphragm and connected to the isolation unit of the S-48 Grass stimulator. Signals from the isometric force transducer were amplified, collected, and digitized with the aid of a computer equipped with a Digidata-1322A A/D interface board (Axon Instruments, Union City, CA, USA). Each hemi-diaphragm preparation was allowed to equilibrate for 20–30 min before starting experiments.

### Inhibition by competition of ^125^I-α-bungarotoxin binding to muscle-type nAChR

To measure the affinity constant of the αC-conotoxin PrXA for the *Torpedo* nAChRs, competition binding assays were performed at equilibrium on 96-well plates by incubating during 4 h, 0.05 μg of *Torpedo* membranes with different concentrations of αC-conotoxin PrXA (0.1 nM to 10 µM) and [^125^I]-α-BgTx (1 nM to 2.5 nM). Non-specific binding was determined in the presence of 1 μM [^125^I]α−BgTx. In equilibrium competition experiments, IC_50_ values were determined by fitting the competition data by the empirical Hill’s equation and converted to K_i_ constants using the following equation (1)^40^:

$$Ki=I{C}_{50}/(1+({\rm{L}}\ast \frac{1}{{\rm{Kd}}}))$$

with a K_d_ for [^125^I]-α−BgTx on muscle-type receptor of 50 pM. To evaluate the protective effect of the aptamers on the αC-conotoxin PrXA-*Torpedo* nAChR interaction, aptamers (0.1 nM to 20 µM) were preincubated 1 h with αC-conotoxin PrXA (1 µM) before the addition to the receptor-[^125^I]-α−BgTx mixture for 4 h. All the reactions were stopped by filtration of the 96-well simultaneously through a GF/C plate pre-soaked in 0.5% polyethylenimine, using a FilterMate harvester (PerkinElmer, France). The filters were washed twice with ice-cold buffer (PBS), dried and the bound radioactivity was counted after the addition of 25 µl of MicroScint 0 per well, by scintillation spectrometry on a TopCount beta counter (PerkinElmer).

### Neutralization of the *in vivo* lethality of αC-conotoxin PrXA by the selected aptamers

Adult male and female *Mus musculus* Swiss mice weighting 18 to 20 g were used to test the lethal effects of αC-conotoxin PrXA. Mice were maintained in groups of six under standard temperature conditions (25 °C) with suitable light conditions (12 h light and dark cycles) and relative humidity (65 ± 5%). The animals were allowed free access to standard laboratory food and water. The experiments were carried out in accordance with the Cameroon Ethical Committee Guidelines (Yaoundé, Cameroon, Ref. N°FWA-IRB00001954) and International (EEC Council Directive 86/609, OJL 358, 1, Dec. 12, 1987; Guide for the Care and Use of Laboratory Animals, U.S. National Research Council, 1996) for the care and use of laboratory animals. All efforts were made to minimize the suffering and number of animals used.

Lethality and latency to death induced by αC-conotoxin PrXA were determined by either intraperitoneal (i.p.) or subcutaneous (s.c.) injection of the toxin at varying concentrations (0.005, 0.008, 0.01, 0.05, 0.1, 0.5, 1 and 1.5 µg/g mouse b.w.). The toxin was dissolved in buffer A solution: 0.09% saline + 5 mM MgCl_2_. The volume injected was 10 µL/g mouse b.w. For toxin neutralization by aptamers *in vivo*, a maximally-effective dose of αC-conotoxin PrXA (0.5 μg/g mouse b.w.) was injected at 0.5 µg/g mouse b.w. i.p. or s.c., followed 1 min later by i.v. administration of various aptamer concentrations into the tail vein. In other experiments, the toxin was incubated in buffer A with varying amounts of aptamers for 2 h at 4 °C (0.5 µg/g mouse b.w. for aptamers A5, B4, D3 and D7, and 0.0625 to 8 µg/g mouse b.w. for aptamers B4 and D7). Each toxin/aptamer mixture was injected i.p. or s.c. at a volume of 10 µL/g mouse b.w. for each group of six Swiss mice (3 males and 3 females). Controls received 0.5 µg/g mouse b.w. of αC-conotoxin PrXA in buffer A solution. For each condition, the general behavior of each animal was observed continuously during 1 h after injection, then intermittently for 4 h and thereafter over a period of 24 h. In addition, the mice were further observed for up to 14 days after the treatment for any sign of toxicity and late latency of death. Any adverse effects, such as hypoactivity, piloerection, salivation and syncope were evaluated immediately after administration of different compounds. Furthermore, anorexia and weight loss were observed and noted.

## Electronic supplementary material

Supplementary Information
