# Supplementary material for: Efficient functional neutralization of lethal peptide toxins in vivo by oligonucleotides
Source: Sci Rep. 2017 Aug 3;7:7202. doi: 10.1038/s41598-017-07554-5 (PMC5543128; doi:10.1038/s41598-017-07554-5)
Supplement: Supplementary file 1 — Supplementary Information [file 41598_2017_7554_MOESM1_ESM.pdf]

Supplementary information

Efficient functional neutralization of lethal peptide toxins *in vivo* by oligonucleotides

Tarek Mohamed Abd El-Aziz, Corinne Ravelet, Jordi Molgo, Emmanuelle Fiore, Simon Pale, Muriel Amar, Sawsan Al-Khoury, Jérôme Dejeu, Mahmoud Fadl, Michel Ronjat, Germain Sotoing Taiwe, Denis Servent, Eric Peyrin and Michel De Waard

SI Materials and Methods

Materials

N-α-Fmoc-L-aminoacid and Wang-Tentagel resin and reagents used for peptide synthesis were obtained from Iris Biotech (Markterdwitz, Germany). Analytical grade quality solvents (acetonitrile (ACN), dimethylformamide (DMF), N-methylpyrrolidone (NMP), trifluoroacetic acid (TFA) were from Acros Organics (Illkirch, France). The 77-nucleotide fluorescent single-stranded DNA (ssDNA) library (general nucleotide sequence: 5'-FAM-GCCTGTTGTGAGCCTCCTGTCGAA-random 30 nucleotides-TTGAGCGTTTATTCTTGTCTCCC-3') and heavier reverse primer were from Eurofins Genomics (Ebersberg, Germany). Fluorescent 5'-labeled forward primer and unlabeled forward primer were from Eurogentec (Liège, Belgium). GeneAmp 10X PCR buffer II and AmpliTaq Gold DNA polymerase were from Applied Biosystems (Foster City, California, USA). HS-(CH2)11-EG6-Biotin was procured from Prochimia (Vallet, France). [^{125}I]α−Bungarotoxin ([^{125}I]α−BgTx) (210–250 Ci × mmol^{-1}) was purchased from PerkinElmer (Courtaboeuf, France).

Chemical syntheses of αC-conotoxin PrXA and its fluorescent analogue

Chemical syntheses of αC-conotoxin PrXA and fluorescent FAM-αC-conotoxin PrXA were performed by the solid-phase method using an automated peptide synthesizer (CEM© Liberty, Orsay, France). Peptide chains were assembled stepwise on 0.24 meq of Fmoc-L-Arg(Pbf)-Wang-Tentagel resin using 0.24 mmol of N-α-fluorenylmethyloxycarbonyl (Fmoc) L-amino-acid derivatives. The following side-chain protecting groups were used: trityl for Cys and Asn, tert-butyl for Ser, Thr, Glu and Asp, Pbf for Arg and tert-butylcarbonyl for Lys. Reagents were at the following concentrations: Fmoc-amino-acids (0.2 M Fmoc-AA-OH in DMF), activator (0.5 M 2-(1H-benzotriazole-1-yl)-1,1,3,3-tetramethyluronium hexafluorophosphate in DMF), activator base (2 M diisopropylethylamine in NMP) and deprotecting agent (5% piperazine/0.1 M 1-hydroxybenzotriazole in DMF), as advised by the PepDriver software (CEM©).

After peptide chain assemblies, resins were treated 4 h at room temperature with a mixture of TFA/water/triisopropylsilane (TIS)/dithiothreitol (DTT) (92.5/2.5/2.5/2.5; v/v/v/v). The peptide mixtures were then filtered and the filtrates were precipitated by adding cold tert-butylmethyl ether. The crude peptides were pelleted by centrifugation (10,000 × *g*, 15 min) and the supernatants were discarded. The peptides were purified by Reverse-Phased High Pressure Liquid Chromatography (RP-HPLC) using a Vydac C18 column (218TP1010, 4 µm, 250 × 100 mm) using a 10–60% ACN linear gradient containing 0.1% TFA. Crude peptides were then oxidized/folded in 0.1 M Tris-HCl buffer at pH 8.3 for 48 h before purification of the folded/oxidized peptides by RP-HPLC using a Vydac C18 column (218TP104, 5 µm, 250 ID × 46 mm L) again with a 10-60% ACN linear gradient with 0.1% TFA. Correct oxidation of the synthesized peptides were checked by MALDI-TOF mass spectrometry.

Aptamer selection against αC-conotoxin PrXA using the CE-SELEX approach

Aptamer selection - CE-SELEX was performed using a 77-nucleotide single-stranded DNA (ssDNA) library containing a randomized region of 30-nt central region flanked by two conserved primer hybridization regions (23-nt at 5' end position and 24-nt at 3' end position). Selection was performed on a Beckman Coulter P/ACE MDQ system (Fullerton, California, USA) with exchangeable UV absorbance and laser-induced fluorescence (LIF) detectors (λ_{ex} 488 nm and λ_{em} 520 nm). The capillary was 60 cm in length (48.5 cm from inlet to detection window) with an inner diameter of 50 µm and an outer diameter of 360 µm (Polymicro Technologies Inc., Phoenix, Arizona, USA). The fused-silica capillaries were conditioned by performing the following washes at 20 psi: 1 M NaOH for 5 min, water for 5 min and TGK buffer (25 mM Tris, 192 mM Glycine, 5 mM KH_2PO_4 (pH 7.34)) for 30 min. The washing process between runs was performed at 20 psi with 1 M NaOH (2 min), water (2 min) and TGK buffer (5 min). DNA library was heated at 80°C for 5 min and left at room temperature for 15 min. αC-conotoxin PrXA (750 nM) was then added to the DNA library for the first round of selection. The mixture was incubated at room temperature for 20 min. For each selection round, DNA sequences, target peptide and TGK buffer were combined in 30 µL total volume. Toxin concentrations were 20 nM, 6 nM and 0.5 nM for rounds 2, 3 and 4, respectively. The equilibrated sample was injected at 0.7 psi for 20 sec and separated under 20 kV voltage. UV detection was used to monitor the separation. During a selection round, the eluate was collected into 300 µL TGK buffer until the unbound DNA peak began to elute. The injection, separation and collection process was repeated five more times for round 1, 10 times for round 2 and 9 times for rounds 3 and 4. Input DNA concentration was determined by absorbance at 260 nm on a Shimadzu UV mini-1240 spectrophotometer with a Tray Cell from Hellma Analytics (Müllheim, Germany). For the first round of selection, the unselected library was used with 40 µM concentration in the incubated sample. Subsequent rounds of selection used collected DNA, amplified and purified from the previous round as the input DNA.

The concentration of input DNA in later selection rounds was 40, 1, 3 and 2.5 μM, respectively. Approximately 10^{12} sequences were introduced into the capillary in the first round of selection.

PCR amplification and single-stranded DNA Production - Single-stranded DNA candidates were generated using a reverse primer and a 5'-FAM-labeled forward primer during each round of the selection procedure. The reverse primer is made heavier with a succession of six C3 links extended with a 5' DNA stretch of 20 nucleotides (5'-ACTGACTGACTGACTGACTA-6C3-GGGAGACAAGAATAAACGCTCAA). During PCR, this six C3 region and the 20-nucleotide stretch cannot be amplified by the Taq DNA polymerase. A PCR product with two strands of unequal length is consequently synthesized. Each strand is then easily purified on a denaturing polyacrylamide gel. All PCR were performed using a Biometra cycler from Labgene (Archamps, France). Master mix was made by combining 886 μL nuclease-free water, 64 μL dNTPs (25 mM of each) (Invitrogen, Cergy Pontoise, France), 200 μL each of forward (5'-FAM-GCCTGTTGTGAGCCTCCTGTCGAA) and heavier reverse primers (10^{-5} M), 240 μL $MgCl_2$ (25 mM), and 200 μL GeneAmp 10X PCR buffer II (500 mM potassium chloride and 100 mM Tris-HCl, pH 8.3). After mixing, 10 μL (5 U/μL) of AmpliTaq Gold DNA polymerase was added. To finish, 200 μL of DNA collected during selection were added. This mixed solution was divided equally over thin-walled tubes that were subjected to PCR. The thermal cycling regime was: initial denaturation for 10 min at 95°C, and then cycling for 60 sec at 95°C, 60 sec at 60°C and 90 sec at 72°C for 20 cycles. After a Nanosep 3K purification (Pall, Washington, New York, USA), the samples, which contained different amounts of amplified products, were resolved on a 12% acrylamide gel at an applied voltage of 300 V. The band corresponding to the library molecular weight and corresponding to selected aptamers was visualized by UV-shadow method at 254 nm, cut and eluted for 1 h at 65°C and 1 h at 4°C, in 1 mL of the extraction buffer (100 mM Tris-HCl (pH 7.4), 500 mM NaCl, 1 mM EDTA). To remove acrylamide, the extracted product was transferred into a Nanosep device, the retention membrane was replaced by glass wool and the system was centrifuged during 10 min at 14,000 rpm. Filtrate was removed, transferred in another native Nanosep 3K device and centrifuged for 90 min at 5,000 rpm at 15°C. The retentate was washed with 100 μL of water and centrifuged again. The amount of single strand was quantified by UV absorbance at 260 nm.

Binding Affinity, cloning and sequencing - Four rounds of selection were performed and the progress of selection was monitored using non-equilibrium capillary electrophoresis of equilibrium mixtures (NECEEM) to estimate the bulk affinity. Capillary conditions (length capillary, preconditioning, migration buffer, voltage, temperature) were identical to those used for the capillary electrophoresis aptamer selection, except for sample preparation. Each sample contained 100 nM of enriched DNA pools and αC-conotoxin PrXA was added to a final concentration ranging from 0 to 85 μM. The volume of peptide was constant in all samples. The enriched library from round 4 was chosen for cloning and sequencing and realized by Biofidal (Vaulx en Velin, France).

Aptamer binding affinity measurement using surface plasmon resonance (SPR)

The binding affinity of the selected aptamer B4 sequence against αC-conotoxin PrXA were analyzed by SPR at 25°C using a Biacore T200 instrument (Biacore, GE Healthcare). Sensor chips were cleaned by UV/ozone treatment (10 min) followed by rinsing with MilliQ water and ethanol. The cleaned gold surfaces were then functionalized according to the following procedure. Firstly, mixed self-assembled monolayers (SAMs) were formed at room temperature by dipping overnight gold sensors in a (9:1) mixture of HS-(CH2)11-EG4-OH and HS-(CH2)11-EG6-Biotin (1 mM total thiol concentration in EtOH). After overnight adsorption, gold sensors were rinsed with EtOH and dried under nitrogen. Conforming to the fluorescence anisotropy experiment, all measurements were conducted in the same TGK buffer containing 0.1% Tween 20. This buffer was filtered in each case and also used as running buffer. Streptavidin (100 ng/mL) was injected (10 µL/min) until saturation of the surface (around 2500 R.U.), following by around 300 RU of 5'-biotinylated B4 aptamer at the same flow rate. Binding experiments were conducted at 50 µL/min by injection of αC-conotoxin PrXA (dissolved in the TGK buffer containing 0.1% Tween 20, injection time: 120 sec, dissociation time: 300 sec, stabilization time: 300 sec) at different concentrations (1 nM to 20 µM) by using a multicycle method. A streptavidin surface, prepared as described above, was used as reference. Curves obtained on the reference surface were deduced from the curves recorded on the recognition one, thereby allowing elimination of refractive index changes owing to buffer effects. The kinetic data were treated independently by using the Bio-Kine software (Biologic, Claix, France) to give the apparent rate constants k_{obs}. The k_{obs} related to the specific association were plotted against the complex concentrations to extract the k_{on} values, while the k_{obs} for the dissociation were averaged to give k_{off}.

SI Supplementary Figures

Supplementary Fig. 1. (**a**) RP-HPLC purification of chemically synthesized αC-conotoxin PrXA. Inset: MALDI-TOF MS of the purified toxin. The primary structure of the toxin is indicated on top, along with the connectivity of the disulfide bridge. O: 4-*trans*-hydroxyproline. (**b**) RP-HPLC purification of the FAM-labeled αC-conotoxin PrXA. Inset: MALDI-TOF MS of the purified FAM-labeled toxin. The primary structure of the toxin is indicated on top, along with the position of the FAM in the sequence.

Supplementary Fig. 2. (**a,b**) Electropherograms of αC-conotoxin PrXA (**a**, first peak), initial ssDNA library (**A**, second peak) and toxin-aptamer complex (**b**, first peak). The injection consisted of 705 µM of αC-conotoxin PrXA, 33.3 µM of ssDNA library and a complex of 50 µM of αC-conotoxin PrXA and 100 nM of ssDNA library. The capillary electrophoresis conditions are: TGK buffer, 0.7 psi/20 sec for the

injection, 20 kV for the separation, 25°C and UV (**a**) & Laser-induced Fluorescence (LiF) detection (**b**). (**c**) Dissociation constant (K_d) of the ssDNA pool to αC-conotoxin PrXA after each round of the CE-SELEX process.

Supplementary Fig. 3. (**a**) Predicted secondary structures of B4 (four structures) and D7 (one structure) (**b**) aptamers (created by Mfold website with the following parameters: 4°C, 100 mM Na^+, 5 mM Mg^{2+}).

Supplementary Fig. 4. Binding of αC-conotoxin PrXA to the biotinylated B4 aptamer immobilized on SPR sensorchip. (**a**) SPR sensorgrams for the interaction between B4 aptamer and αC-conotoxin PrXA used at different concentrations (from 160 nM to 20 µM). (**b**) k_{obs} during association for the interaction as a function of toxin concentration. (**c**) k_{obs} during dissociation for the interaction as function of toxin concentration.

Supplementary Fig. 5. (**a**) Amino acid and disulfide bridge pairing of waglerin 1. (**b**) Dose-response curve illustrating mice death percentage by various concentrations of waglerin 1. Two routes of administrations were used: i.p. and s.c. (**c**) Shortening of the latency of death induction as a function of waglerin 1 concentration.

Supplementary Table 1. Phenotype variation in mice challenged with αC-conotoxin PrXA or waglerin 1. D/T = dead/treated mice; None = No toxic symptoms during the observation period; mortality latency = time to death (in minute) after the intraperitoneal injection. αC-conotoxin PrXA or waglerin 1 were administered to group of male and female mice. Mice in each group were carefully examined for any signs of toxicity (behavioural changes or mortality) for 1 h. Control group received distilled water (10 mL/kg, intraperitoneal).

Supplementary Table 2. Aptamer sequences of the clones isolated after four rounds of selection using CE-SELEX. Primers are in bold script. Aptamer sequences chosen for affinity measurements and functional evaluation are in red. The sequence of scramble aptamers used in various controls are also shown. S1: scramble with same primer sequences, S2: D7 scramble sequence, and S3: random scramble.

Supplementary Table 3. Dissociation constants (K_d) of the selected aptamer sequences against FAM-αC-conotoxin PrXA after random selection of each aptamer family from sequences supplied in Supplementary Table 2. Fluorescence anisotropy change was measured to determine K_d values. S1 scramble sequence did not induce any anisotropy change.

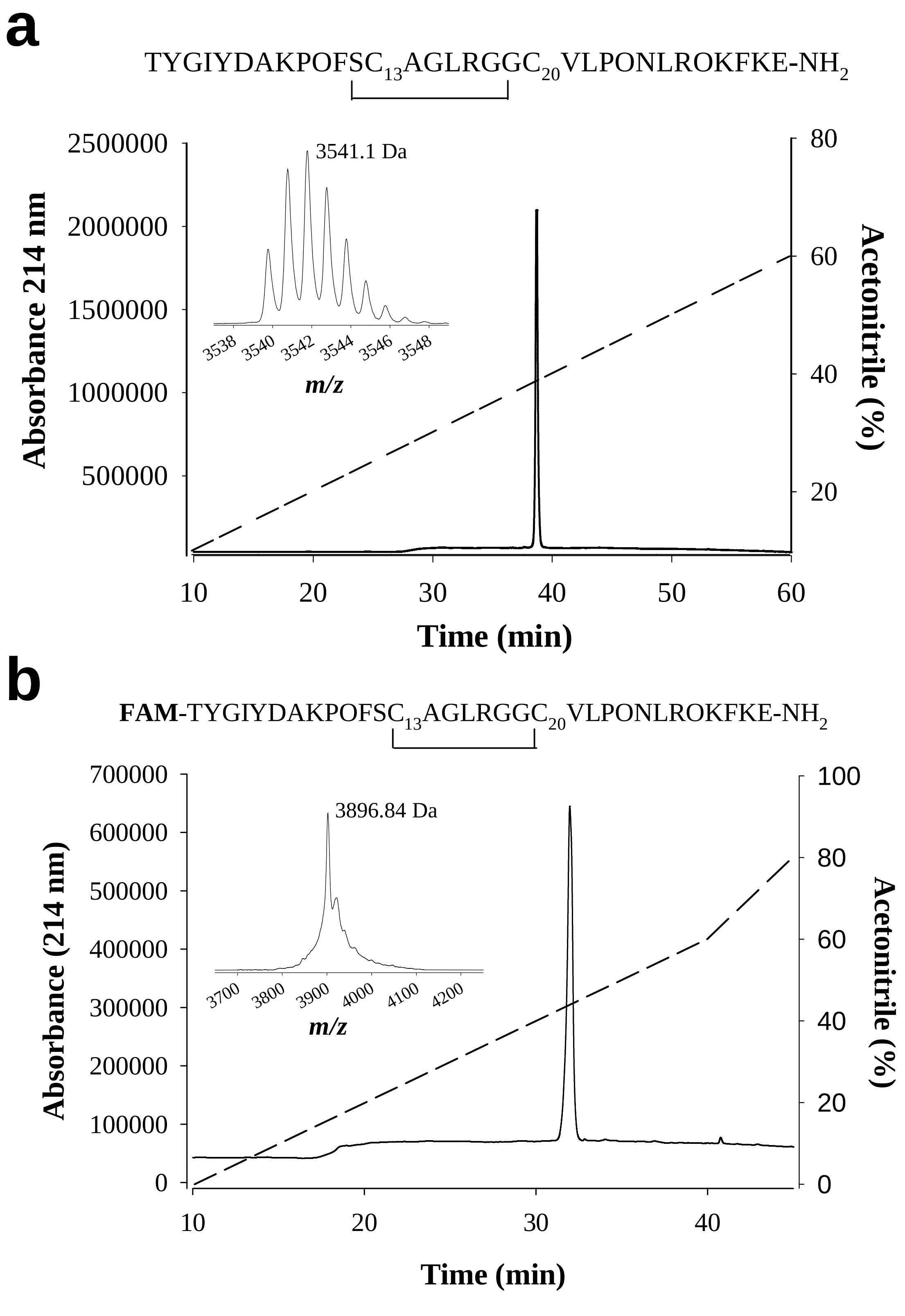

Supplementary Fig. 1

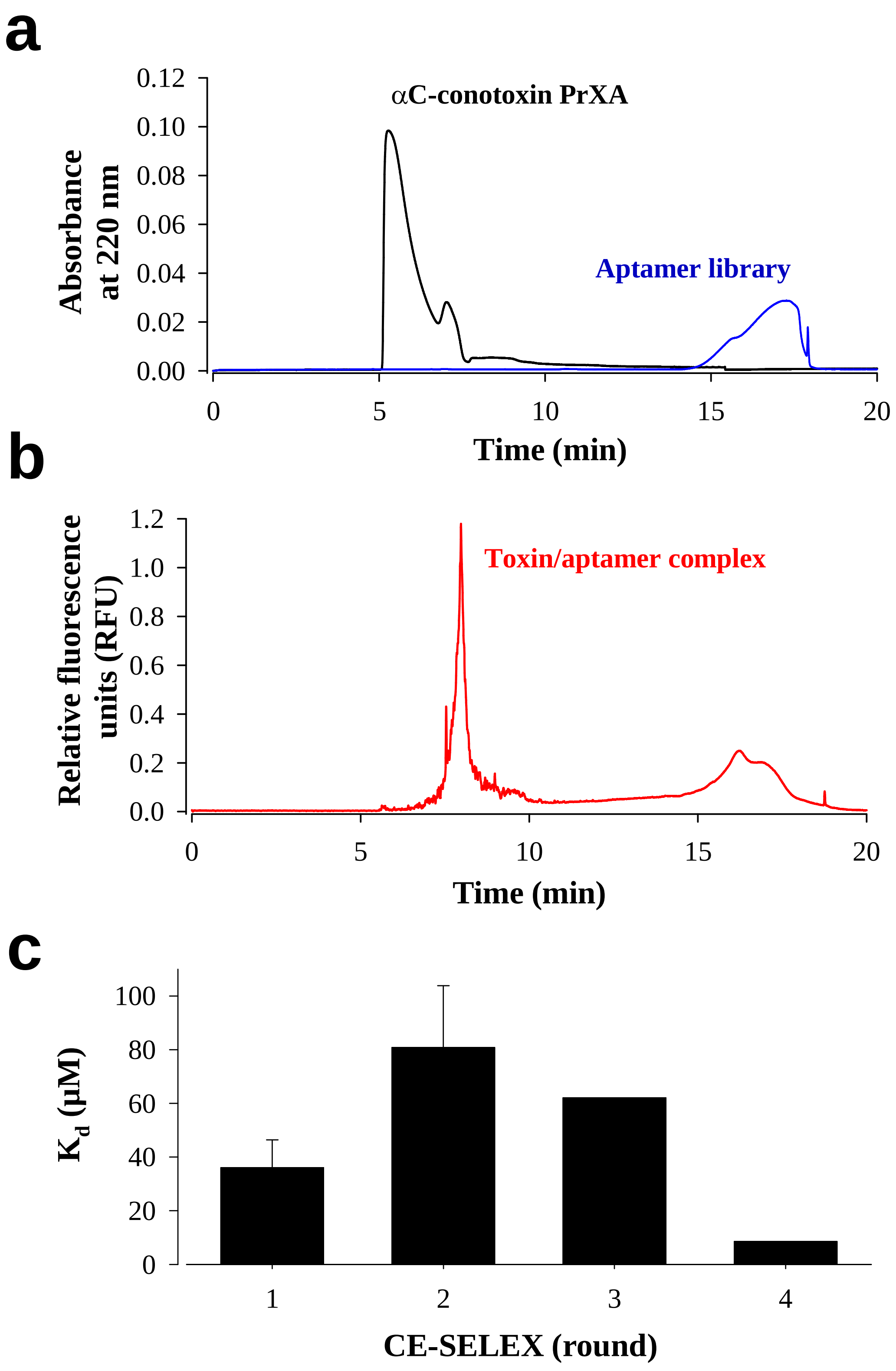

Supplementary Fig. 2

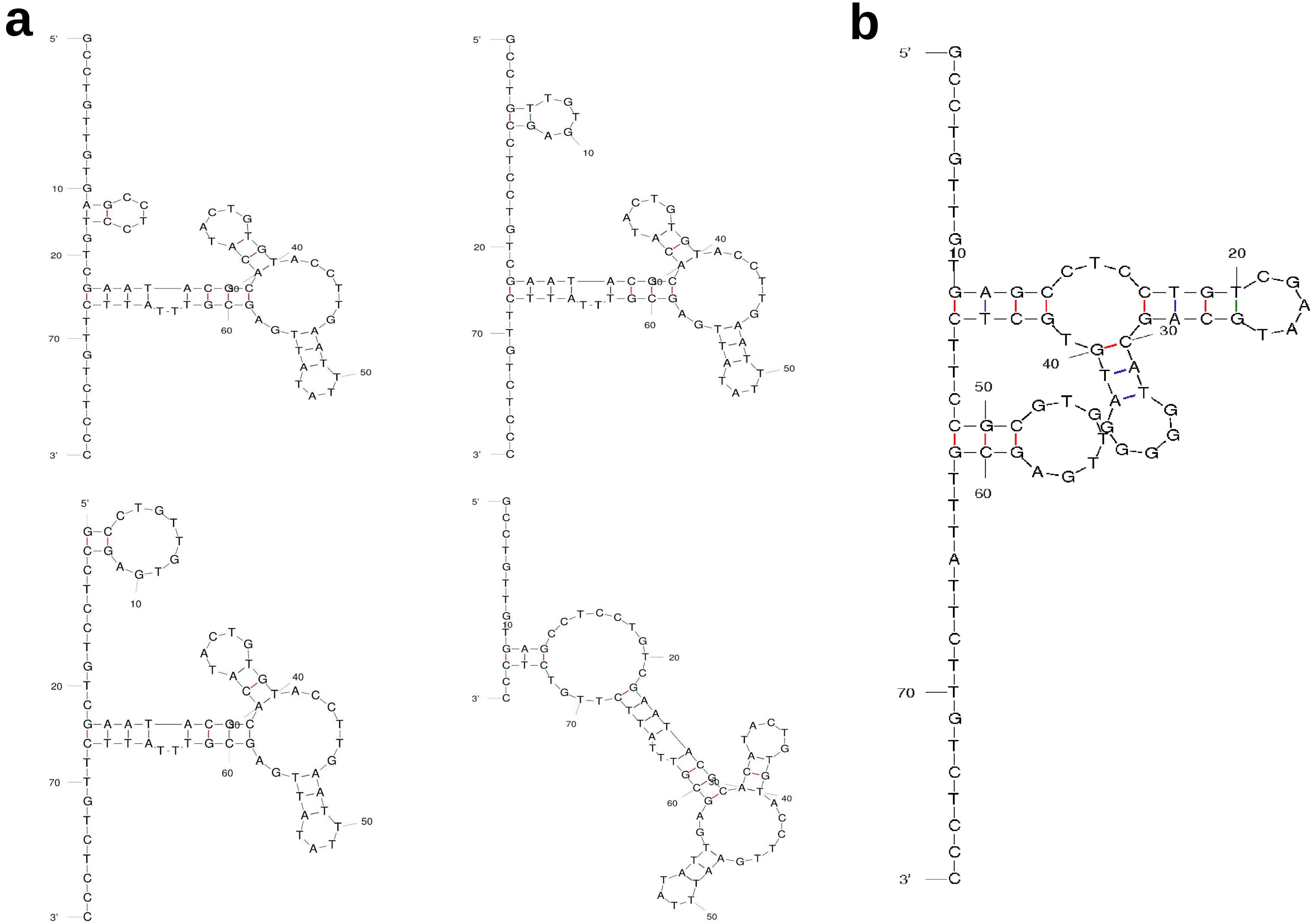

Supplementary Fig. 3

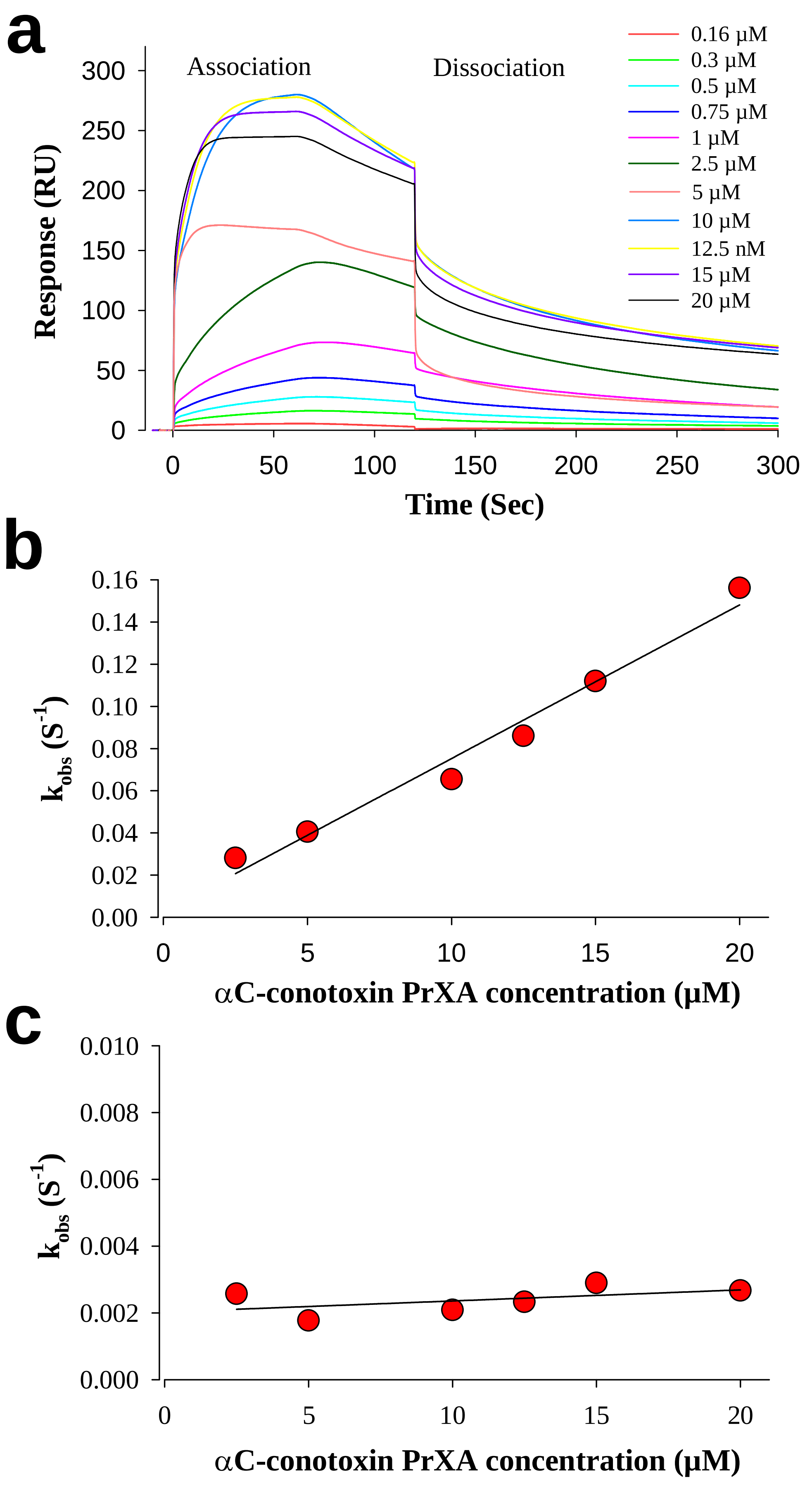

Supplementary Fig. 4

a

Waglerin 1

GGKPDLRPC$_9$HPPC$_{13}$HYIPRPKPR

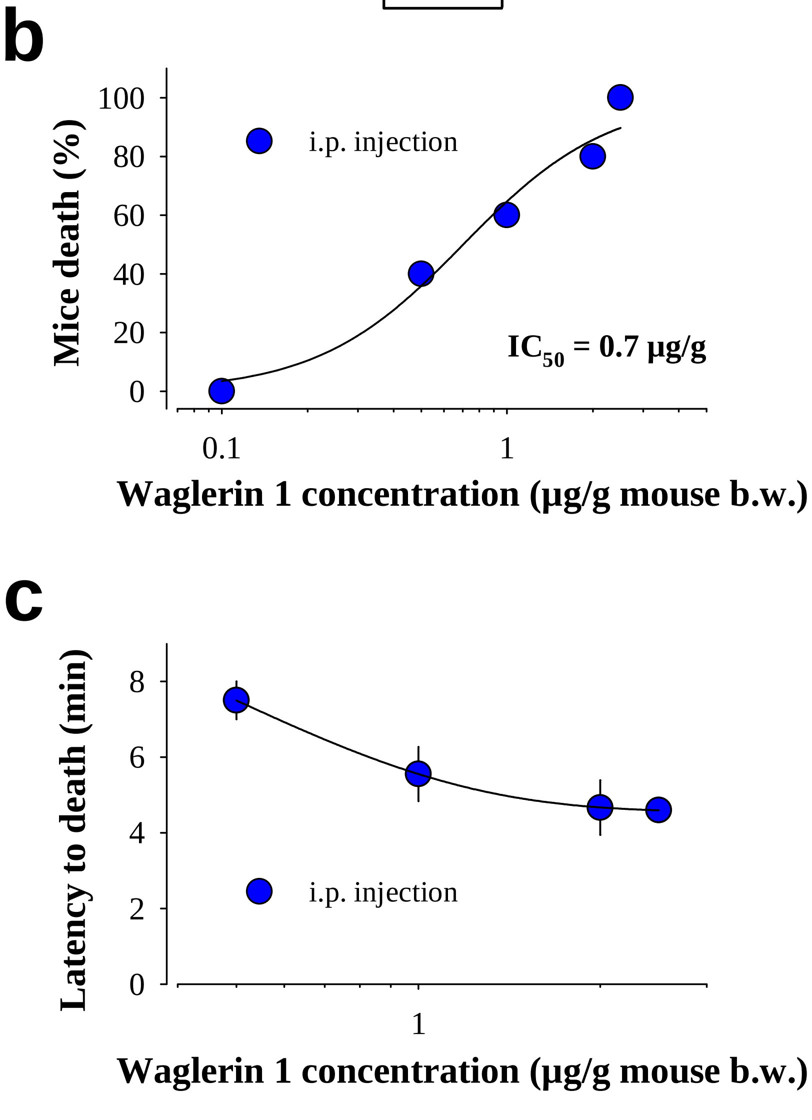

Supplementary Fig. 5

Supplementary Table 1.

Treatments	Concentration (µg)	Sex	D/T	Mortality latency (min)	Toxic symptoms
Control	–	Male<br>Female	0/3<br>0/3	– –<br>– –	None<br>None
αC-conotoxin PrXA	0.1	Male<br>Female	3/3<br>3/3	≥1, ≤4<br>≥1, ≤4	Hypoactivity, asthenia, tachypnea, tremors, loss of the righting reflex, myoclonus, exophthalmos, salivation and syncope
	0.5	Male<br>Femelle	3/3<br>3/3	≥1, ≤3<br>≥1, ≤3	Hypoactivity, piloerection, salivation, tachypnea, tremors, loss of the righting reflex, myoclonus, exophthalmos, salivation and syncope
	1	Male<br>Female	3/3<br>3/3	≥1, ≤3<br>≥1, ≤3	Hypoactivity, piloerection, salivation, Tachypnea, , tremors, loss of the righting reflex, myoclonus, exophthalmos, salivation and syncope
	1.5	Male<br>Female	3/3<br>3/3	≥1, ≤2<br>≥1, ≤2	Piloerection, salivation, tachypnea, tremors, loss of the righting reflex, myoclonus, exophthalmos, salivation and syncope
Waglerin 1	10	Male<br>Female	2/3<br>2/3	≥7, ≤8<br>≥7, ≤8	Piloerection, tachypnea, tremors, loss of the righting reflex, myoclonus, and exophthalmos
	20	Male<br>Female	3/3<br>3/3	≥5, ≤9<br>≥5, ≤9	Piloerection, tachypnea, tremors, loss of the righting reflex, myoclonus and exophthalmos
	40	Male<br>Female	3/3<br>3/3	≥4, ≤8<br>≥4, ≤8	Piloerection, tachypnea, tremors, loss of the righting reflex, myoclonus and exophthalmos
	50	Male<br>Female	3/3<br>3/3	≥1, ≤4<br>≥1, ≤4	Piloerection, tachypnea, tremors, loss of the righting reflex, myoclonus and exophthalmos

Supplementary Table 2.

Cloning Name	Sequence (5' to 3')	Number of bases
**Family 1**		
A3	**GCCTGTTGTGAGCCTCCTGTCGAA**ATAGGGACATTCCGCAGCTCTACGCCGCTT**TTGAGCGTTTATTCTTGTCTCCC**	77
A5	**GCCTGTTGTGAGCCTCCTGTCGAA**GTGCAGGTCTATACAGGACAGTCTTCTGAT**TTGAGCGTTTATTCTTGTCTCCC**	77
D3	**GCCTGTTGTGAGCCTCCTGTCGAA**ATCGGTCGTATAGGGTCGATTTGGTCGGCA**TTGAGCGTTTATTCTTGTCTCCC**	77
B6	**CCTGTTGTGAGCCTCCTGTCGAA**TTTGATGGCATAACATTCGGATCGTGCCTG**TTGAGCGTTTATTCTTGTCTCCC**	77
**Family 2**		
C10	**GCCTGTTGTGAGCCTCCTGTCGAA**TACGAGCCCGCTGGGACTTGCATTAGTGC**TTTGAGCGTTTATTCTTGTCTCC**	77
B4	**GCCTGTTGTGAGCCTCCTGTCGAA**TACGCACATACTGTGTACCTTGAATTTATA**TTGAGCGTTTATTCTTGTCTCCC**	77
D2	**GCCTGTTGTGAGCCTCCTGTCGAA**TAGATGCCTTTTAATATGCCATGATGAAGG**TTGAGCGTTTATTCTTGTCTCCC**	77
A4	**GCCTGTTGTGAGCCTCCTGTCGAA**AATGCTGTTGTTTGAGTATCAATCAGACCG**TTGAGCGTTTATTCTTGTCTCCC**	77
**Family 3**		
D7	**GCCTGTTGTGAGCCTCCTGTCGAA**TGCAGCATGGGGGATGTGCTCTTCCGCGTG**TTGAGCGTTTATTCTTGTCTCCC**	77
B3	**GCCTGTTGTGAGCCTCCTGTCGAA**CCGTAGATGCGGGGATGCCAGTCTTGCTTA**TTGAGCGTTTATTCTTGTCTCCC**	77
**Scrambles**		
S1	**GCCTGTTGTGAGCCTCCTGTCGA**AGGGGATGGACCCCTACTTCATTCGCCCGCC**TTGAGCGTTTATTCTTGTCTCCC**	77
S2	TGGACGTTCGGGCATCCTGGTTATGGGTTAGTGACACGTGGTCGGCCTTCTGGTATCTCTGTCATTCGCCTTGCCCT	77
S3	ACTGCTGATGACGACTACTGGTGACGACTACTGCTGATGACGACTACTGCTGATGACGACTCTGATGACTGACTACTG	77

Supplementary Table 3.

Aptamer Cloning Name	Kd (nM)	Standard deviation (±)
Aptamer B4	120.3	21.3
Aptamer D3	122.0	25.3
Aptamer A5	184.4	69.7
Aptamer D7	237.5	30.6
Aptamer A4	246.1	66.6
Aptamer B3	> 5000	> 3000
Aptamer S1	-	-
